# Pelvic Diameters and Their Association With Maternal Body Mass Index, Parity, and Delivery Outcomes: A Cross-Sectional Study

**DOI:** 10.7759/cureus.77573

**Published:** 2025-01-17

**Authors:** Lana Lacevic Mulahasanovic, Lejla Dervišević, Almir Fajkić, Mirna Rakocevic Selimovic, Aida Dizdarevic Aljovic, Altaira Jazic Durmisevic, Ilvana Hasanbegovic, Zurifa Ajanović, Aida Sarac-Hadzihalilovic, Edina Lazović Salčin, Amela Dervišević

**Affiliations:** 1 Gynecology and Obstetrics, Clinical Center University of Sarajevo, Sarajevo, BIH; 2 Anatomy, University of Sarajevo, Faculty of Medicine, Sarajevo, BIH; 3 Pathophysiology, University of Sarajevo, Faculty of Medicine, Sarajevo, BIH; 4 Emergency Medicine, Clinical Center University of Sarajevo, Sarajevo, BIH; 5 Pathology, University of Sarajevo, Faculty of Medicine, Sarajevo, BIH; 6 Human Physiology, University of Sarajevo, Faculty of Medicine, Sarajevo, BIH

**Keywords:** bmi, delivery outcome, multiparous, pelvic diameters, primiparous

## Abstract

Background

In addition to age, body mass index (BMI), abdominal circumference, and parity, measuring the mother's pelvic diameters is a non-invasive, cost-effective method that can assist gynecologists in determining the optimal management of labor. Our study aimed to examine the associations between maternal age, pelvic diameters, BMI, abdominal circumference, and parity with delivery outcomes and investigate differences in pelvic diameters in relation to maternal age, BMI, delivery outcomes, parity, and episiotomy.

Materials and methods

The observational, cross-sectional study included 108 pregnant women in the active phase of labor who were admitted to the Gynecological Clinic at the Clinical Center University of Sarajevo. During admission, maternal data were registered: age, body height, body weight, abdominal circumference, and BMI. Using a pelvinometer, pelvic diameters were recorded: interspinous diameter (DS), intertrochanteric diameter (DT), intercristal diameter (DC), and external conjugate (CE). The Anterior Pelvic Index (API) was calculated by dividing the DS by the participants' height and multiplying the result by 100. Data were analyzed using SPSS Statistics for Windows, Version 17 (Released 2008; SPSS Inc., Chicago, United States).

Results

Women who underwent cesarean section were significantly older compared to those with spontaneous vaginal delivery. A significant correlation was observed between maternal age, BMI, and delivery outcomes. Obese women had significantly higher DT compared to women with normal or overweight BMI. Primiparous and multiparous women differed significantly in CE, while other pelvic diameters did not differ. Women with episiotomy had significantly lower DS and CE diameters compared to those without episiotomy during vaginal delivery.

Conclusion

Maternal age, BMI, and pelvic diameters are significant delivery outcome determinants; our findings suggest that these parameters deserve to be included in delivery outcome assessment as they provide substantial information in the journey of achieving personalized delivery care and decision-making.

## Introduction

The manner of delivery, whether vaginal or cesarean, is influenced by various maternal and fetal factors, including maternal anthropometric characteristics. Maternal anthropometric measurements, including weight, height, body mass index (BMI), pelvic diameters, and maternal age, play an important role in identifying maternal and fetal health status during pregnancy [1]. These measurements are important for predicting possible complications, helping in clinical decision-making, and providing an outcome for safe delivery processes. These factors are major determinants of short- and long-term maternal and neonatal outcomes [2,3]. Since maternal measurements are critical for preventing complications and optimizing care, as well as safeguarding the physical well-being of both mother and child [4], it becomes imperative to understand the link between these maternal measurements and the mode of delivery [4].

A general physical examination during pregnancy includes an assessment of constitution, height, and body mass; an examination of the skeletal system; measurements of the pelvis; and the presence of edema. The pelvinometer measures the outer dimensions of the pelvis. When these are known, they provide information as to the state of the bony part of the pelvis, which is another important value when predicting vaginal birth [5]. Pelvic measurement is important for predicting labor and safe childbirth. The dimensions and shape of the maternal pelvis directly influence the ability of the fetus to pass through the birth canal during delivery [6,7]. Proper evaluation of the anatomy of the pelvis helps healthcare providers to identify potential risks, like cephalopelvic disproportion (CPD). This assessment is particularly important for decision-making regarding labor management, including the trial of labor, the need for instrumental delivery, or cesarean section [8]. Pelvic measurements from clinical and radiological examinations are a window into predicting morbidities; therefore, they are important in alleviating maternal and neonatal morbidity and predicting delivery outcomes [9].

Another significant factor influencing birth mode is maternal age. Age-related biological changes and social and medical circumstances can affect pregnancy progression and delivery [10]. Younger mothers, particularly teenagers, are at higher risk of complications such as preterm labor, low birth weight, and pregnancy-induced hypertension, often due to physical immaturity or limited access to prenatal care [11]. Conversely, advanced maternal age (35 years and above) is associated with increased risks of conditions like gestational diabetes, preeclampsia, chromosomal abnormalities, and the need for operative deliveries [12].

The objective of this study was to evaluate whether these maternal factors (age, BMI, and pelvic diameters) can predict the mode of delivery, thus allowing the identification of women at higher risk for cesarean section or those likely to have a vaginal delivery.

## Materials and methods

This observational, cross-sectional study was conducted between September and November 2024 on women admitted to the maternity ward of the Gynecological Clinic of the Clinical Centre University of Sarajevo. All women participated voluntarily, with prior verbal explanation. Women consented to participate in the study during pain-free periods between contractions. The authors guarantee the confidentiality and privacy of data throughout the research work.

The study included 108 women aged over 18 years who voluntarily agreed to participate and satisfied the inclusion criteria. The inclusion criteria were the cephalic presentation of the fetus and women in the active phase of labor (cervical dilation of more than 4 cm with regular contractions, i.e., one to three contractions in 10 minutes). Women who had a high-risk pregnancy or relevant previous surgical intervention (previously more than two cesarean sections) that could have affected the outcome of labor were not included in the study. Also, pregnancies with expected fetal comorbidities were not included in this study.

On admission, each woman's height and weight were routinely measured using a digital weight and height scale (Model No.: FM-S120), based on which BMI was calculated according to the formula: "weight in kg/height in m^2^." Height was measured without shoes, and the women were wearing underwear during weight measurement. Based on BMI values, women were classified into three categories according to the WHO classification: normal weight 18.5-24.9 kg/m²; overweight 25-29.9 kg/m²; and obese 30-34.8 kg/m².

Determining the external dimensions of the pelvis was done using a pelvinometer, which provided insight into the condition of the bony part of the pelvis. When measuring the external measurements of the pelvis, the pregnant woman lay on her back with her legs slightly bent at the hips and knees, while for the measurement of the external conjugate, the pregnant woman lay on her side with the lower leg bent at the hip and knee and the upper leg extended over it. The interspinous diameter was measured as the distance between the two anterior superior iliac spines. Intercristal diameter was measured as the distance between the two most distant points on the iliac crest. The intertrochanteric diameter was measured as the distance between the two greater trochanters of the femur. The external conjugate was measured as the distance between the middle of the upper edge of the pubic symphysis and the spinous process of the fifth lumbar vertebra. The Anterior Pelvic Index (API) was determined by dividing the interspinous diameter by the height of women and multiplying the outcome by 100 [13]. Abdominal circumference (AC) was measured with a soft tape measure at the most prominent point of the abdomen, corresponding to the level of the umbilicus.

Women with slow-progressing labor or irregular uterine contractions were given an oxytocin drip. Cardiotocographic monitoring was performed throughout the labor. An experienced gynecologist decided to conduct a cesarean section after taking into account all monitored parameters.

Statistical analysis

Data were analyzed using the SPSS Statistics for Windows, Version 17 (Released 2008; SPSS Inc., Chicago, United States).. The Kolmogorov-Smirnov test assessed the normality of continuous variables. Results are expressed as means (M) and standard deviations (SD) for normally distributed continuous variables or as medians (Me) and interquartile ranges (IQR) for non-normally distributed variables. Differences were tested using the student's t-test for normally distributed continuous variables. In contrast, the Mann-Whitney U test was applied for two independent groups with non-normal distributions and the Kruskal-Wallis test for three groups. Qualitative data are presented as absolute values (n) and percentages (%) and were analyzed using the chi-square test (χ2). Statistical significance was set at p<0.05.

## Results

The average age of the women included in the study was 31.4 ± 5.9 years; the youngest was 20, and the oldest was 44. Of the total number of women (n=108), 47 (43.5%) were primiparous, while 61 (56.5%) were multiparous. In 57 (52.8%), the pregnancy ended in a spontaneous vaginal birth, while in 51 (47.2%) women, the outcome of the pregnancy was a cesarean section. The median body height (cm) was 167.5 (164.0 - 171.0). The median body weight (kg) measured before delivery was 83.0 (75.0 - 89.75), the abdominal circumference (cm) measured before delivery was 108.0 (103.0 - 112.0), and BMI (kg/m^2^) values ​​calculated before delivery were 29.4 (26.9 - 31.64) (Table 1).

**Table 1:** Characteristics of the overall study cohort Results are presented as mean ± SD, median and interquartile range (25 - 75 percentile) %: percentages; n: number of subjects

Parameters	Study cohort (n=108)
Age (years)	31.4 ± 5.9
Min.	20
Max.	44
Height (cm)	167.5 (164.0 - 171.0)
Weight (kg)	83.0 (75.0- 89.75)
Body mass index (kg/m^2^)	29.4 (26.9- 31.64)
Abdomen circumference (cm)	108.0(103.0 - 112.0)
Maternal parity - n (%)	
Primiparous	47 (43.5)
Multiparous	61 (56.5)
Delivery type- n (%)	
Vaginal spontaneous	57 (52.8)
Cesarean	51 (47.2)

The age of women in whom the section was performed (33.1 ± 5.7 years) was statistically significantly higher (p=0.004) compared to those who had spontaneous vaginal births (29.9 ± 5.7 years). A statistically significant correlation was found between the age of the women and the delivery mode (p=0.011).

Women whose pregnancy ended with spontaneous vaginal delivery had significantly (p=0.045) higher BMI values ​​compared to those whose pregnancy ended by section (30.12 kg/m^2^ (28.0 - 31.96) vs. 27.77 kg/m^2^ (26.4 - 31.4)). A statistically significant (p=0.017) correlation was found between BMI values and delivery mode.

No statistically significant difference was found in body height (p=0.106), body weight (p=0.301), and abdominal circumference measured before delivery (p=0.095) between women whose pregnancy ended in spontaneous vaginal delivery and those with section. Also, no significant correlation (p=0.275) was found between the number of previous births and the delivery outcome.

A total of 22 (38.6%) women who delivered vaginally were primiparous, while 35 (61.4%) patients were multiparous. Among women who delivered by cesarean section, 25 (49%) were primiparous, and 26 (51%) were multiparous. There was no statistically significant difference in the number of deliveries and mode of delivery (p=0.275) (Table 2).

**Table 2:** Maternal characteristics by delivery type Results are presented as: mean ± SD, or median and interquartile range (25 - 75 percentile) %: percentages; BMI: body mass index; n: number of subjects; p: probability; *-p<0.05

Parameters	Vaginal spontaneous delivery (n=57)	Cesarean delivery (n=51)	p
Age (years)	29.9 ± 5.7	33.1 ± 5.7	0.004*
Age (groups) - n (%)			
≤ 28	28 (49.1)	11 (21.6)	0.011*
29-35	16 (28.1)	20 (39.2)	
≥ 36	13 (22.8)	20 (39.2)	
Height (cm)	167.0 (162.5 - 170.0)	168.0 (164.0 - 173.0)	0.106
Weight (kg)	85.0 (77.5 - 89.5)	80.0 (72.0 - 90.0)	0.301
Body mass index (kg/m^2^)	30.12(28.0 - 31.96)	27.77(26.4 - 31.4)	0.045
BMI groups - n (%)			
Normal weight (18.5-24.9)	1 (1.8)	8 (15.7)	0.017*
Overweight (25.0-29.9)	51 (47.2)	25 (49.9)	
Obese (≥ 30)	48 (44.4)	18 (35.3)	
Abdomen circumference (cm)	109.0 (105.0 - 112.0)	107.0 (102.0 - 111.0)	0.095
Maternal parity - n (%)			
Primiparous	22 (38.6)	25 (49.0)	0.275
Multiparous	35 (61.4)	26 (51.0)	

No statistically significant difference was found in the values ​​of the pelvic diameters (intercristal diameter (DC), intertrochanteric diameter (DT), interspinous diameter (DS), external conjugate (CE), and Anterior Pelvic Index (API)) and the way the delivery ended, vaginally or by cesarean section (Table 3).

**Table 3:** Differences in pelvic diameters and API between women categorized base on delivery type Results are presented as the median and interquartile range (25 - 75 percentile) n: number of subjects, p: probability; DC: intercristal diameter; DT: intertrochanteric diameter; DS: interspinous diameter; CE: external conjugate; API: Anterior Pelvic Index

Pelvic parameters	Vaginal delivery (n=57)	Cesarean delivery (n=51)	p
DC (cm)	28.0 (27.0 - 29.5)	28.0 (27.0 - 29.0)	0.416
DT (cm)	35.0 (31.0 - 36.0)	34.0 (30.0 - 35.0)	0.190
DS (cm)	26.0 (25.0 - 30.0)	27.0 (25.0 - 30.0)	0.958
CE (cm)	19.0 (19.0 - 20.5)	20.0 (18.0 - 20.0)	0.813
API	16.25 (15.15 - 18.75)	15.9 (14.9 - 18.4)	0.301

The external conjugate value differed statistically between primiparous and multiparous women (p=0.043), while the DC, DT, and DS values ​​did not differ significantly between primiparous and multiparous women (Table 4).

**Table 4:** Differences in pelvic diameters and API between participants categorized based on the number of deliveries Results are presented as: median and interquartile range (25-75 percentile) n: number of subjects; p: probability. DC: intercristal diameter; DT: intertrochanteric diameter; DS: interspinous diameter; CE: external conjugate; API: Anterior Pelvic Index

Pelvic parameters	Primiparous (n=47)	Multiparous (n=61)	p
DC (cm)	28.0 (27.0 - 29.0)	28.0 (27.0 - 30.0)	0.250
DT (cm)	35.0 (30.0 - 36.0)	35.0 (30.5 - 36.0)	0.913
DS (cm)	26.0 (25.0 - 30.0)	28.0 (25.5 - 30.0)	0.154
CE (cm)	19.0 (18.0 - 20.0)	20.0 (19.0 - 21.0)	0.043
API	15.76 (14.94 - 17.65)	16.85 (15.2 - 18.75)	0.172

No statistically significant differences were found in pelvic parameters (DC, DT, DS, DE) and API values ​​between women in all age groups (Table 5).

**Table 5:** Differences in pelvic diameters and API between women categorized based on age Results are presented as: median and interquartile range (25-75 percentile) n: number of women; p: probability; DC: intercristal diameter; DT: intertrochanteric diameter; DS: interspinous diameter; CE: external conjugate; API: Anterior Pelvic Index

Pelvic parameters	Age ≤ 28 (n=39)	Age ≤29≥35 (n=36)	Age ≥ 36 (n=33)	p
DC (cm)	28.0 (27.0 - 29.0)	27.0 (26.25 - 29.0)	28.0 (27.0 - 29.5)	0.553
DT (cm)	35.0 (30.0 - 36.0)	35.0 (30.25 - 35.0)	35.0 (31.0 - 36.0)	0.529
DS (cm)	26.0 (25.0 - 30.0)	26.0 (25.0 - 30.0)	29.0 (26.0 - 30.0)	0.202
CE (cm)	20.0 (18.0 - 20.0)	19.5 (18.0 - 20.75)	20.0 (19.0 - 21.0)	0.467
API	15.88 (15.15 - 18.4)	15.84 (14.97 - 18.2)	16.85 (15.14 - 18.6)	0.534

Obese women had statistically significantly higher (p=0.013) intertrochanteric diameter compared to those with normal body weight and compared to overweight women (p=0.035). The values ​​of other pelvic diameters (DC, DS, and CE), as well as the API values, ​​did not differ significantly among the women categorized based on BMI (Table 6).

**Table 6:** Differences in pelvic diameters and API between women categorized based on BMI Results are presented as: median and interquartile range (25-75 percentile). *p<0.05-compared to women with normal body weight; ♦p<0.05-compared to overweight women n: number of women; p: probability; DC: intercristal diameter; DT: intertrochanteric diameter; DS: interspinous diameter; CE: external conjugate; API: Anterior Pelvic Index

Pelvic parameters	Normal weight (n=9)	Overweight (n=51)	Obese (n=48)	p
DC (cm)	27.0 (26.0 - 29.0)	28.0 (27.0 - 29.0)	28.0 (27.0 - 30.0)	0.577
DT (cm)	31.0 (28.0 - 35.0)	34.0 (30.0 - 36.0)	35.0*^♦^ (32.5 - 36.75)	0.013^*^ 0.035^♦^
DS (cm)	26.0 (25.5 - 31.0)	28.0 (26.0 - 30.0)	26.0 (25.0 - 30.0)	0.180
CE (cm)	19.0 (18.0 - 20.0)	20.0 (18.0 - 20.0)	20.0 (19.0 - 21.0)	0.094
API	15.29 (15.03 - 19.2)	16.47 (15.29 - 18.75)	15.82 (14.97 - 17.89)	0.301

Women whose pregnancies ended in spontaneous vaginal birth with an episiotomy had statistically significantly lower values ​​of interspinous diameter (p=0.013) and external conjugate (p=0.019) compared to those whose pregnancies ended in spontaneous vaginal birth without an episiotomy. Other pelvic parameters (DC and DT), as well as API, did not differ significantly between women with spontaneous vaginal birth with an episiotomy and those without an episiotomy (Table 7).

**Table 7:** Differences in pelvic diameters and API between women with vaginal delivery with and without episiotomy Results are presented as: median and interquartile range (25-75 percentile) n: number of women; p: probability; *p<0,05; DC: intercristal diameter; DT: intertrochanteric diameter; DS: interspinous diameter; CE: external conjugate; API-Anterior Pelvic Index

Pelvic parameters	Vaginal delivery with episiotomy (n=23)	Vaginal delivery without episiotomy (n=34)	p
DC (cm)	27.0 (27.0 - 29.0)	28.0 (27.0 - 30.0)	0.216
DT (cm)	35.0 (30.0 - 36.0)	35.0 (31.75 - 36.0)	0.818
DS (cm)	25.0 (25.0 - 29.0)	29.0 (26.0 - 30.5)	0.013*
CE (cm)	19.0 (18.0 - 20.0)	20.0 (19.0 - 21.0)	0.019*
API	15.76 (15.0 - 17.14)	17.14 (15.3 - 18.8)	0.06

## Discussion

The majority of births in the world end vaginally. Vaginal birth is the preferred method of ending a pregnancy because it carries significantly fewer risks for the mother and the baby [14]. However, in certain situations, vaginal delivery requires conversion to a cesarean section. The cesarean section is one of the most common surgical procedures in obstetrics that saves both mother and child [15,16]. Many health centers around the world, especially in rural areas, are not equipped to perform cesarean sections. Therefore, it is important to assess specific maternal parameters that may indicate whether a vaginal or cesarean delivery should be performed. Long distances, as well as poor local transportation, can lead to obstructions in labor and uterine rupture [17]. Our study aimed to examine the associations between maternal age, pelvic diameters, BMI, abdominal circumference, and parity with delivery outcomes, as well as to investigate differences in pelvic diameters concerning maternal age, BMI, delivery outcomes, parity, and episiotomy.

In our study, women who had a cesarean section were significantly older compared to women who had a vaginal delivery (33.1 ± 5.7 vs. 29.9 ± 5.7 years; p=0.011). This can be due to a combination of physiological, obstetric, and social factors. Labor in older patients is often prolonged or not adequately progressing, a condition known as dystocia [18]. This is becoming more frequent due to weaker contractions of the uterus or structural changes in the reproductive system [19].

In some healthcare systems, older maternal age is considered a high-risk category. This leads to a lower threshold for performing C-sections, even in borderline cases, to minimize potential risks [20]. Our results agree with Rydahl et al. and Richards et al., who found a strong connection between women’s age and the cesarean section [21,22]. Dystocia during labor may affect its duration; however, the literature contains conflicting results [23]. Greenberg et al. found that dystocia in older women prolongs the first and second stages of labor, while Zaki et al. found that labor progresses more rapidly in older women, indicating that the uterus still has sufficient strength to contract and complete labor [24,25]. Recent studies have shown that the risk of cesarean section is two to seven times more common in women over 40 and that this incidence increases with age [26]. Veenstra et al. stated that there are four best predictors of unplanned cesarean section in women over 40 years old: high BMI, no prior vaginal births, spontaneous start of labor, and number of days of cervical priming [27].

In our study, women whose pregnancy ended by vaginal mode had significantly higher BMI, compared with those who had a cesarean section (p=0.045). At our clinic, it is a common practice to aim for vaginal delivery in women with higher body weight as long as it is safe for the mother and the child. The reason for this lies in the fact that obese women are more likely to develop cellulitis after a cesarean section, which requires a more extended hospital stay and recovery [28]. On the other hand, obese women require more significant amounts of anesthetic when undergoing general anesthesia, which increases the risk of giving birth to a depressed child [29].

Women with a higher BMI have a larger pelvic capacity, which makes it easier for the baby to pass through the birth canal. The dimensions of the pelvis are thought to be one of the factors determined by the constitution of the body and play an important part in determining the mode of delivery [30]. In addition to mechanical factors, pregnant women who are overweight or obese have been shown to have increased levels of estrogen and leptin, hormones that can reduce the elasticity of pelvic ligaments and contractility of the uterus [31]. Enhanced soft tissue compliance may support the natural progression of labor, thus facilitating vaginal birth [32,33]. In contrast, some studies suggest that the emergency cesarean section delivery rate is more often in obese women, which could be attributed to the more frequent deconditioning in women with a high BMI [34,35].

In this study, it has been shown that women with higher BMI tend to have a higher intertrochanteric distance compared to women with standard BMI values (p=0.013) and compared to women who are overweight (p=0.035). Other pelvic parameters (DC, DS, CE) did not differ among different groups of women according to BMI values. Bone is a dynamic tissue that remodels in response to mechanical forces [36]. The pelvis bones, including the iliac crest and femoral neck, adapt to the increased fat deposits, which leads to the outward displacement of hip bones [37]. As a compensatory mechanism to support the increased body weight and greater fat, the greater trochanters of the femur move away, which results in increased DT measurements [38].

We found no correlation between pelvic parameters and mode of delivery. A larger external conjugate was registered in multiparous women compared to primiparous. The external conjugate of the pelvis, also known as the pelvic conjugate, is a critical measurement in obstetrics, representing the distance between the pubic symphysis and the sacral promontory. This measurement plays a key role in determining the adequacy of the birth canal and is crucial in assessing whether a woman can deliver vaginally [39].

One of the key hormones secreted during pregnancy is relaxin, which plays a key role in softening the ligaments of the sacroiliac joint and the pubic symphysis. In this way, relaxin facilitates passage through the birth canal [40,41]. The effect of relaxin is more pronounced in women who have given birth multiple times because the pelvic ligaments do not return to their original state of tightness as they did before pregnancy. This may lead to permanent changes in pelvic width and be the reason why the external conjugate is greater in women who have given birth multiple times than in women who have given birth for the first time. Repeated pregnancies and deliveries can lead to pelvic remodeling, which alters the shape and size of the pelvic cavity, including the external conjugate [40]. In our study, we did not find a significant relationship between the method of delivery and the number of previous births or whether the woman was primiparous or multiparous (p=0.275). The lack of statistical significance in delivery mode between multiparous and primiparous women can be attributed to a variety of factors, including sample size and power limitations, clinical and medical practice standardization, confounding variables, and the complexity of biological and psychosocial factors influencing delivery decisions [42].

Our study observed statistically smaller values of external conjugate and interspinous diameter in women who delivered vaginally but with the help of an episiotomy compared to women whose delivery was also completed vaginally but without an episiotomy. Radnja et al. found a significant frequency of episiotomy in primiparous women with shorter perineal size and anogenital distance [43]. A smaller interspinous diameter and smaller external diameter are significant risk factors for obstructed labor because they limit the available space for the baby to descend and rotate properly [30]. This study has several limitations that should be acknowledged. First, the cross-sectional design limits the ability to establish causal relationships between maternal anthropometric measurements and delivery outcomes. Second, the relatively small sample size may reduce the generalizability of the findings to broader populations. Additionally, the study was conducted at a single institution, which may not reflect variations in clinical practices across different healthcare settings. Future studies should consider these factors in larger, more diverse populations to validate and expand upon these findings.

## Conclusions

Our findings suggest that maternal age, BMI, and pelvic diameters play a significant role in determining delivery outcomes, with obesity being associated with larger DT. While pelvic parameters, such as DC, DT, DS, DE, and API, did not differ significantly between delivery methods or age groups, certain factors, such as parity and episiotomy, influenced specific pelvic measurements. These results highlight the importance of considering maternal anthropometric measurements and pelvic diameters in the assessment of delivery outcomes, offering valuable information for personalized care and decision-making during pregnancy and labor.

## References

[1] Frederick IO, Williams MA, Sales AE, Martin DP, Killien M (2008). Pre-pregnancy body mass index, gestational weight gain, and other maternal characteristics in relation to infant birth weight. Matern Child Health J.

[2] Moghaddam Tabrizi F, Saraswathi G (2012). Maternal anthropometric measurements and other factors: relation with birth weight of neonates. Nutr Res Pract.

[3] Kretzer DC, Matos S, Von Diemen L (2020). Anthropometrical measurements and maternal visceral fat during first half of pregnancy: a cross-sectional survey. BMC Pregnancy Childbirth.

[4] Korhonen U, Taipale P, Heinonen S (2013). Assessment of bony pelvis and vaginally assisted deliveries. ISRN Obstet Gynecol.

[5] Awonuga AO, Merhi Z, Awonuga MT, Samuels TA, Waller J, Pring D (2007). Anthropometric measurements in the diagnosis of pelvic size: an analysis of maternal height and shoe size and computed tomography pelvimetric data. Arch Gynecol Obstet.

[6] Steer P, Flint C (1999). ABC of labour care: physiology and management of normal labour. BMJ.

[7] Heckman JD, Sassard R (1994). Musculoskeletal considerations in pregnancy. J Bone Joint Surg Am.

[8] Beck R, Malvasi A, Cinnella G, Van De Velde M (2021). Pelvic Anatomy, Cephalopelvic Disproportion, Intrapartum Sonography and Neuraxial Analgesia. Intrapartum Ultrasonography for Labor Management.

[9] Maharaj D (2010). Assessing cephalopelvic disproportion: back to the basics. Obstet Gynecol Surv.

[10] Hochler H, Lipschuetz M, Suissa-Cohen Y (2023). The impact of advanced maternal age on pregnancy outcomes: a retrospective multicenter study. J Clin Med.

[11] de la Calle M, Bartha JL, Lopez CM, Turiel M, Martinez N, Arribas SM, Ramiro-Cortijo D (2021). Younger age in adolescent pregnancies is associated with higher risk of adverse outcomes. Int J Environ Res Public Health.

[12] Cabrera Ramos SG (2023). Obstetric complications and advanced maternal age. Rev Peru Ginecol Obstet.

[13] Guerrero M, Ocampo J, Zapata M, Yépez E (2016). Determination of anterior pelvis index (api) to predict a narrow pelvis in adolescent girls. International Journal of Morphology.

[14] Lagrew DC, Low LK, Brennan R (2018). National Partnership for maternal safety: consensus bundle on safe reduction of primary cesarean births-supporting intended vaginal births. Obstet Gynecol.

[15] Vogel JP, Betrán AP, Vindevoghel N (2015). Use of the Robson classification to assess caesarean section trends in 21 countries: a secondary analysis of two WHO multicountry surveys. Lancet Glob Health.

[16] Kheir AEM, Ali RBA, Ahmed MAM (2016). Comparison of neonatal outcome associated with elective caesarean section versus planned vaginal delivery in a low-risk obstetric population. Int J Curr Res.

[17] Lowe NK (2007). A review of factors associated with dystocia and cesarean section in nulliparous women. J Midwifery Womens Health.

[18] Herstad L, Klungsøyr K, Skjærven R, Tanbo T, Forsén L, Åbyholm T, Vangen S (2016). Elective cesarean section or not? Maternal age and risk of adverse outcomes at term: a population-based registry study of low-risk primiparous women. BMC Pregnancy Childbirth.

[19] Witter FR, Caulfield LE, Stoltzfus RJ (1995). Influence of maternal anthropometric status and birth weight on the risk of cesarean delivery. Obstet Gynecol.

[20] Kissler K, Hurt KJ (2023). The pathophysiology of labor dystocia: theme with variations. Reprod Sci.

[21] Richards MK, Flanagan MR, Littman AJ, Burke AK, Callegari LS (2016). Primary cesarean section and adverse delivery outcomes among women of very advanced maternal age. J Perinatol.

[22] Rydahl E, Declercq E, Juhl M, Maimburg RD (2019). Cesarean section on a rise-does advanced maternal age explain the increase? A population register-based study. PLoS One.

[23] Winata IGS, Putri TIADWP, Pradnyandari AAAV (2024). Diagnosis and management of labor dystociaaccording to the Friedman curve. European Journal of Medical and Health Sciences.

[24] Greenberg MB, Cheng YW, Sullivan M, Norton ME, Hopkins LM, Caughey AB (2007). Does length of labor vary by maternal age?. Am J Obstet Gynecol.

[25] Zaki MN, Hibbard JU, Kominiarek MA (2013). Contemporary labor patterns and maternal age. Obstet Gynecol.

[26] Rademaker D, Hukkelhoven CW, van Pampus MG (2021). Adverse maternal and perinatal pregnancy outcomes related to very advanced maternal age in primigravida and multigravida in the Netherlands: a population-based cohort. Acta Obstet Gynecol Scand.

[27] Veenstra J, Cohen Z, Korteweg FJ (2024). Unplanned cesarean sections in advanced maternal age: a predictive model. Acta Obstet Gynecol Scand.

[28] Thornburg LL, Linder MA, Durie DE, Walker B, Pressman EK, Glantz JC (2012). Risk factors for wound complications in morbidly obese women undergoing primary cesarean delivery. J Matern Fetal Neonatal Med.

[29] Casati A, Putzu M (2005). Anesthesia in the obese patient: pharmacokinetic considerations. J Clin Anesth.

[30] Li J, Lou Y, Chen C (2023). Predictive value of MRI pelvimetry in vaginal delivery and its practicability in prolonged labour-a prospective cohort study. J Clin Med.

[31] Leblanc DR, Schneider M, Angele P, Vollmer G, Docheva D (2017). The effect of estrogen on tendon and ligament metabolism and function. J Steroid Biochem Mol Biol.

[32] Wuntakal R, Kaler M, Hollingworth T (2013). Women with high BMI: should they be managed differently due to antagonising action of leptin in labour?. Med Hypotheses.

[33] Simpson ER (2003). Sources of estrogen and their importance. J Steroid Biochem Mol Biol.

[34] Melzer K, Schutz Y, Soehnchen N (2010). Effects of recommended levels of physical activity on pregnancy outcomes. Am J Obstet Gynecol.

[35] Sebire NJ, Jolly M, Harris JP (2001). Maternal obesity and pregnancy outcome: a study of 287,213 pregnancies in London. Int J Obes Relat Metab Disord.

[36] Irving D, Hinkley J, Marquart M (2019). The relationship between BMI and stability of intertrochanteric fracture following low-energy falls. A retrospective cohort study. Geriatr Orthop Surg Rehabil.

[37] Sanz-Salvador L, García-Pérez MÁ, Tarín JJ, Cano A (2015). Bone metabolic changes during pregnancy: a period of vulnerability to osteoporosis and fracture. Eur J Endocrinol.

[38] Siccardi M, Valle C, Di Matteo F, Angius V (2019). A postural approach to the pelvic diameters of obstetrics: the dynamic external pelvimetry test. Cureus.

[39] Goldsmith LT, Weiss G (2009). Relaxin in human pregnancy. Ann N Y Acad Sci.

[40] Petersen LK, Vogel I, Agger AO, Westergård J, Nils M, Uldbjerg N (1995). Variations in serum relaxin (hRLX-2) concentrations during human pregnancy. Acta Obstet Gynecol Scand.

[41] Widelock T, Denney J, Brost B (2023). Pregnancy and Parturition: The Physical and Physiological Changes and Their Pathologies. Post-maternity Body Changes.

[42] Khatony A, Soroush A, Andayeshgar B, Saedpanah N, Abdi A (2019). Attitude of primiparous women towards their preference for delivery method: a qualitative content analysis. Arch Public Health.

[43] Radnia N, Khansari S, Jiriaei N, Hosseini SA, Salemi L, Hamoon M (2022). The relationship between perineal size and episiotomy during delivery. J Med Life.

